# Metaviromics reveals a high diversity of viruses belonging to the *Caliciviridae* family in seal feces

**DOI:** 10.1093/ve/veag029

**Published:** 2026-05-07

**Authors:** Marion Desdouits, Julien Schaeffer, Cécile Le Mennec, Michèle Gourmelon, Françoise S Le Guyader

**Affiliations:** Ifremer, MASAE, rue de l'île d'Yeu, 44311 Nantes cedex 03, France; Ifremer, MASAE, rue de l'île d'Yeu, 44311 Nantes cedex 03, France; Ifremer, MASAE, rue de l'île d'Yeu, 44311 Nantes cedex 03, France; Ifremer, DYNECO, 1625 route de Sainte-Anne, CS 10070, 29280 Plouzané, France; Ifremer, MASAE, rue de l'île d'Yeu, 44311 Nantes cedex 03, France

**Keywords:** *Caliciviridae,* norovirus, sapovirus, vesivirus, salovirus, virus discovery, seal virus

## Abstract

Investigating potential zoonotic viruses in animal reservoirs is crucial to anticipate viral emergence. Seals can represent large populations of coastal mammals with unknown consequences on the microbiological quality of their surrounding environment. To assess this, we conducted a metaviromics analysis of feces collected from two species of seals in the North-Western Atlantic (Saint-Pierre et Miquelon archipelago). We focused on the *Caliciviridae* family, which regroups several genera with viruses infecting humans and other mammals, including marine mammals, but none identified in seals (*Phocidae*). Among the assembled sequences identified as *Caliciviridae*, there were four known genera (norovirus, sapovirus, vesivirus, and salovirus) and unknown, distantly related viruses. Complete or nearly-complete genomes could be assembled for each genus. Norovirus and sapovirus sequences from seals were diverse and likely represent several new genogroups or genotypes. Seal vesivirus formed a monophyletic group, representing a potential new species related to the canine vesivirus. Salovirus, which are fish viruses, were likely diet-derived, like the distant sequences which exhibited the hallmarks of caliciviruses and were more closely related to fish and reptile viruses. In conclusion, seals are a reservoir for a large diversity of *Caliciviridae*, some related to norovirus or sapovirus genotypes known to infect humans, and their impact on the quality of coastal water or shellfish should be further assessed. This study expands the knowledge on *Caliciviridae* genetic diversity and circulation in marine mammals.

## 1. Introduction

In the recent past, the emergence and pandemic spread of zoonotic viruses in the human population has proven the importance of monitoring animal reservoirs, including wild animals (Keusch et al. 2022, Sikkema and Koopmans 2026). Indeed, most viral families known to infect humans also comprise viruses infecting animals, especially mammals. Among these viral families, the *Caliciviridae* comprise 11 genera, 7 of which are known to infect mammals—including humans—(lagovirus*,* norovirus, nebovirus, recovirus, sapovirus, valovirus, and vesivirus), 2 infect birds (bavovirus, nacovirus), and the other 2 fish (minovirus and salovirus) (Vinjé et al. 2019). In addition, unclassified members of this family were reported in fish, birds, amphibians, and reptiles (Shi et al. 2016, Wille et al. 2018, Vinjé et al. 2019). This viral family is thus very diverse, both phylogenetically and in terms of host range. It also regroups important human pathogens, with the norovirus and sapovirus causing acute gastroenteritis in humans (Bányai et al. 2018).

*Caliciviridae* are small, non-enveloped viruses with a positive-strand RNA genome of 7.5 to 8.5 kb enclosed within a 30–40 nm icosahedral capsid. Their genome encodes (i) a non-structural polyprotein that is proteolytically cleaved into the proteins necessary for the genome replication, including the protease and the RNA-dependent RNA polymerase (RdRp), (ii) a major capsid protein, VP1, forming most of the viral particle through the self-assembly of 90 dimers, and (iii) a minor capsid protein, VP2, also present at a low stoichiometry in the particle, where it could stabilize the viral capsid and/or mediate entry in host cells (Conley et al. 2019, Desselberger 2019). These viral proteins are encoded in 2 to 3 open-reading frames, depending on the genus. Additional open reading frame (ORF) were seldom confirmed, apart from the ORF4 in the murine norovirus, encoding a virulence factor (Vinjé et al. 2019).

*Caliciviridae* are transmitted through direct contacts with infected individuals or their secretions such as feces, vomitus, or saliva (de Graaf et al. 2016, Ghosh et al. 2022). Fecally-contaminated water or foods can also serve as a transmission route, at least for humans (de Graaf et al. 2016). Norovirus and sapovirus are responsible for large outbreaks of gastroenteritis in the human population and are the most studied members of the family. They are divided into multiple genogroups and genotypes, with those infecting humans circulating globally (Oka et al. 2015, de Graaf et al. 2016). Besides, recombination events within genotypes and antigenic pressure cause the frequent emergence of new dominant norovirus strains that become pandemic (de Graaf et al. 2016). Yet, the reservoir for these emerging strains is not known, which has led to the hypothesis that animal norovirus might contribute (Villabruna et al. 2019). While there can be low genetic distances between some animal norovirus genotypes and human ones, and frequent contacts (with pigs or dogs for instance), no zoonotic transmission events were confirmed up to now (Villabruna et al. 2019). Indeed, a natural host restriction seems to be at play within the *Caliciviridae*, that may be due, in part, to receptor recognition (Zakhour et al. 2010, Vinjé et al. 2019, Yang et al. 2019). Yet, several reverse-zoonosis events, where animals were infected with human norovirus, were described, which confirms that host restriction is not absolute (Villabruna et al. 2019). Moreover, vesivirus of marine mammals display a wide host range and interspecies transmission, exemplified with a confirmed case of human infection (Smith et al. 1998, Sosnovtsev et al. 2017).

Marine mammals can be infected with viruses classified in various genera of the *Caliciviridae* family and likely represent a reservoir for unknown members of this family. Indeed, norovirus strains identified in harbor porpoise (Graaf et al. 2017) and California sea lions (Teng et al. 2018) could not be assigned to any known genogroup. Pinnipeds (*Pinnipedia*) regroup three families of coastal mammals, the *Odobenidae* (walrus), *Otarinae* (sea lions and fur seals), and *Phocidae* (true seals) (Berta and Churchill 2012). They are aquatic carnivores living within the coastal area, sometimes close to or exploited by human populations, favoring possible cross-species transmission. Some norovirus, sapovirus, or vesivirus were identified in walrus, sea lion, or fur seals (Smith et al. 1998, Kluge et al. 2016, Teng et al. 2018, Martínez-Puchol et al. 2022, Prado et al. 2025). In *Phocidae*, to our knowledge, only one recent study reported a small vesivirus sequence in seals from the Caspian sea (Karamendin et al. 2024).

Recent studies using high throughput sequencing have extended the knowledge on *Caliciviridae* host range and diversity in different host taxa (Kluge et al. 2016, Shi et al. 2016, Wille et al. 2018, Reuter et al. 2023), including Pinnipeds (Kluge et al. 2016, Teng et al. 2018, Martínez-Puchol et al. 2022, Karamendin et al. 2024). We recently reported a study of the bacteriome and virome of seal feces collected from the two main species of *Phocidae* in France, i.e. grey seals (*Halichoerus grypus*) and harbor seals (*Phoca vitulina*, also named common seals) (Godino Sanchez et al. 2024). Here, we focus on a subset of these samples, collected in a French overseas island, Saint-Pierre et Miquelon (SPM), off the Canadian eastern coast in the North-Western Atlantic. We undertook a deeper analysis of the *Caliciviridae* sequences obtained on to better characterize the diversity of this important viral family in the two species of seals, and evaluate the zoonotic risk they may pose in SPM.

## 2. Material and methods

### 2.1. Sampling

Fecal samples from seals were collected in SPM archipelago in 2019 and 2020 as described previously (Godino Sanchez et al. 2024). Briefly, individual feces were sampled with a sterile spatula, frozen at −20°C on site, and sent to the Ifremer microbiology laboratory. A total of 22 samples were analyzed here, 8 from grey seals, 12 from harbor seals, and 2 from seals of undetermined species (Table 1).

**Table 1:** List of samples with sampling date and seal species.

**Sample ID**	**Sampling date**	**Species (vernacular)**	**Species (scientific)**
FPh85	16 May 2019	Grey seal	*Halichoerus grypus*
FPh86	16 May 2019	Grey seal	*H. grypus*
FPh107	06 May 2019	Harbor seal	*Phoca vitulina*
FPh110	06 May 2019	Harbor seal	*P. vitulina*
FPh111	06 May 2019	Harbor seal	*P. vitulina*
FPh112	05 November 2019	Undetermined	Undetermined
FPh113	05 November 2019	Undetermined	Undetermined
FPh114	16 July 2019	Harbor seal	*P. vitulina*
FPh115	16 July 2019	Harbor seal	*P. vitulina*
FPh116	16 July 2019	Harbor seal	*P. vitulina*
FPh117	08 August 2019	Harbor seal	*P. vitulina*
FPh119	22 June 2020	Grey seal	*H. grypus*
FPh120	22 June 2020	Grey seal	*H. grypus*
FPh121	22 June 2020	Grey seal	*H. grypus*
FPh122	22 June 2020	Grey seal	*H. grypus*
FPh123	22 June 2020	Grey seal	*H. grypus*
FPh124	22 June 2020	Grey seal	*H. grypus*
FPh125	22 June 2020	Harbor seal	*P. vitulina*
FPh126	22 June 2020	Harbor seal	*P. vitulina*
FPh127	22 June 2020	Harbor seal	*P. vitulina*
FPh128	22 June 2020	Harbor seal	*P. vitulina*
FPh129	22 June 2020	Harbor seal	*P. vitulina*

### 2.2. Sample processing and nucleic acid extraction

Feces samples were processed as published previously for virome analysis (Strubbia et al. 2019). Briefly, 10% suspension of feces in PBS were prepared and homogenized by vortexing. Viral particles were detached from suspended material using pyrophosphate (10 mM final concentration) and sonication (Bandelin UD2200 with cup-horn adaptor, 3× 1 min., maximum power). Solids were pelleted by centrifugation at 8000 × g for 20 min. The supernatant was collected, pH adjusted to 7, and viral particles were concentrated by incubating with polyethylene-glycol (PEG 8000) followed by centrifugation at 11 000 × g for 20 min at 4°C. The PEG pellet was resuspended in 2 ml of warm glycin buffer, filtered up to 0.45 μm using serial syringe filters (Sartorius Minisart), and remaining free nucleic acids were digested using OmniCleave endonuclease (Lucigen). Nucleic acids were extracted with the NucliSens kit (BioMerieux) with 10 ml lysis buffer, 50 μl magnetic silica, and using the eGEN-UP© device (BioMerieux) following the manufacturer’s instructions. Nucleic acids were eluted in 100 μl elution buffer, DNA was digested using the TURBO DNAse (Ambion) and the viral RNA purified using the RNA Clean and Concentrator kit (Zymo Research).

As a positive control, one seal feces (1 g) was spiked with 200 μl of a 10% suspension of a human stool sample positive for norovirus GII.17[P17] and treated alongside the other seal feces samples.

### 2.3. Library preparation and sequencing

Libraries were prepared from the purified RNA and a negative control (sterile RNase-free water) as published (Godino Sanchez et al. 2024). Complementary DNA was synthetized for each sample in triplicates using the Superscript II RT (Thermo) and random hexamers (Thermo), and ultrasonically fragmented (M220 ultrasonicator, MA Covaris). The second strand DNA was synthetized for each replicate and the library finalized using the NEBNext® Ultra™ II RNA Library Prep Kit for Illumina (NEB). Paired 2× 150 bp reads were generated using an Illumina NextSeq 500. Raw data are available in the European Nucleotide Archive database (Study: ERP149173; Samples from ERR11734880 to ERR11734912 and ERR13601248 to ERR13601276).

### 2.4. Bioinformatics analyses

An in-house Nextflow pipeline was used as previously described (Bonny et al. 2021). For each sample, reads from the three replicate libraries were merged to assemble longer contigs (Schaeffer et al. 2023). Low quality reads were trimmed using fastp with a quality threshold at 25 (Chen et al. 2018). Clean reads were deduplicated using CD-hit and mapped on the Silva RNA database to remove ribosomal RNAs. *De novo* assembly was then performed using metaSPAdes (v3.14.0) with kmer lengths 21, 33, 55, 77, 99 (Nurk et al. 2017). For two samples (FPh86, FPh115), assembly was also carried out with MEGAHIT using kmer lengths 21, 33, 55, 77, 99, 127 (Li et al. 2015). Post-process reads were mapped using Bowtie2 v2.3.0 (Langmead and Salzberg 2012) on the metaSPAdes contigs to evaluate the coverage, excluding multi-mapped reads. Contigs were identified using BLASTn with an e-value of 10^−5^, and DIAMOND (Buchfink et al. 2015) with an e-value of 10^−3^, on the NCBI non-redondant database (nr). When both approaches retrieved a hit, the BLASTn one was kept. Taxonomic identification was done using the Entrez direct tool, the taxid allowed to extract information at a defined taxonomic level. Contigs matching a member of the *Caliciviridae* family were retrieved and further classified using the Norovirus Typing Tool 2.0 (Kroneman et al. 2011). Genus assignation was based on both BLASTn/DIAMOND and Norovirus Typing tool results. Abundance expressed as reads per million (rpm) was calculated using the number of reads per genus and the total number of trimmed and deduplicated reads per sample. The heatmap and bubble plots were produced using Prism 10.0.0 (GraphPad Softwares). For two samples (FPh86, FPh115), minority variants were identified using the Bowtie2 mapping results and the Ivar variants tool version 1.2.2 on a Galaxy server. In some instance, the pipeline yielded several overlapping contigs matching the same hit in GenBank, but not assembled together. In this case, we manually fused the contigs and used the new sequence to map all clean and deduplicated reads using Bowtie2. If the resulting mapping, visualized using Tablet 1.21.02.08 (Milne et al. 2013), was homogeneous and covered the whole sequence evenly with > 2 reads, the consensus sequence was exported using the Ivar consensus tool version 1.2.2 in Galaxy, and used for phylogenetic analyses.

### 2.5. Genomic organization and phylogeny

Long contigs belonging to the *Caliciviridae* family (raw contigs) were manually cured based on the Bowtie2 mapping to remove extremities covered by less than 3 reads, to produce clean contigs. Geneious Prime 2024.0.7 was used to recover reference sequences from the NCBI Nucleotide database, to detect ORF in references and contigs, extract their sequences, translate them into protein sequences using the standard genetic code, and identify conserved motifs. Nucleotide and/or protein sequences from the clean contigs and the references were aligned using T-coffee tool (version 11.0.8_1) on a Galaxy server, with the muscle algorithm. IQ-tree tool on Galaxy was used to test for the best evolutionary models using the TESTNEW option and the Akaike information criterion (AIC), and to construct maximum-likelihood phylogenetic trees with 1000 fast bootstrap replicates. The Conserved domain database and CD-search tool (NCBI) were used to identify conserved domains within the polyprotein of fish viruses and a putative ORF4 of seal vesivirus. Simplot++ (Samson et al. 2022) was used to plot sequence similarity across the genome based on alignments of seal norovirus complete genomes with reference sequences from GII[GII.P], GIV[GVI.P], GVIII[GII.P28], GIX[GII.P15], and GVII genogroups/P-groups.

### 2.6. Controls and statistics

The positive control containing the norovirus GII.17[P17] yielded the whole genome of that strain (7571b, with 571.013 reads), indicating that extractions, libraries, and sequencing were adequately performed. Importantly, norovirus reads assigned to GII.17[P17] were only detected in the positive control and in sample FPh117 where 5 reads were assembled into a 388b contig that was considered a contaminant from the control, probably during sequencing. To avoid analyzing possible contaminants or falsely assembled contigs, only contigs gathering more than 100 reads were considered for phylogenetic analyses.

## 3. Results

### 3.1. Overall diversity of *Caliciviridae* in seal feces

**Figure 1:**
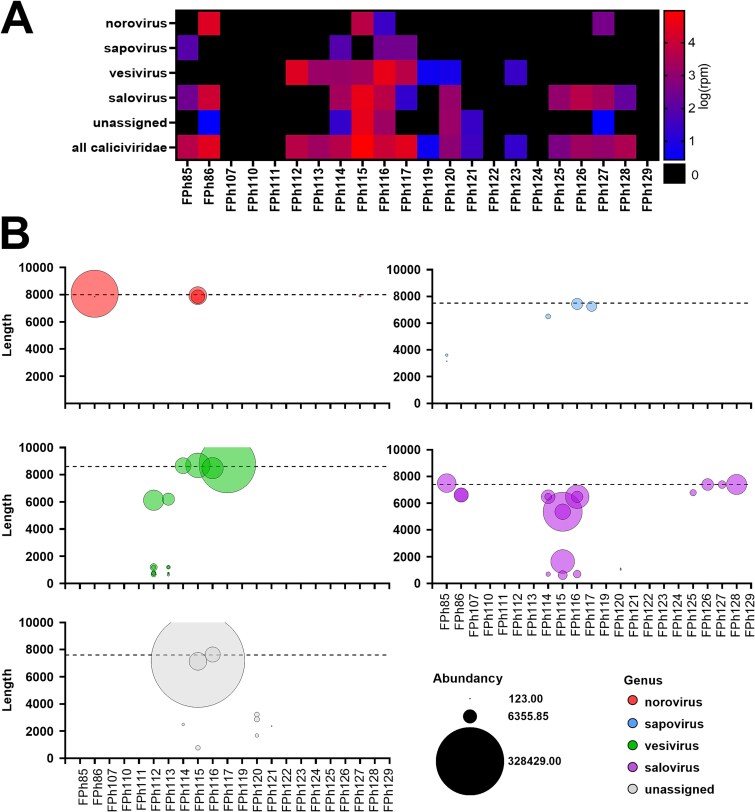
Reads and assembled contigs assigned to *Caliciviridae* belong to four different genera. (A) Heatmap of the number of reads per million clean reads (rpm) assembled into contigs assigned to norovirus, sapovirus, vesivirus, and salovirus, as well as unassigned *Caliciviridae*, in each sample. (B) Length (y-axis) and abundancy (bubble size) of contigs assembled from at least 100 reads in the different samples (x-axis), per genus (bubble color). The length of whole genomes is depicted with a dotted line.

Taxonomic assignation of the assembled contigs allowed to identify calicivirus sequences in 16 samples out of 22 (Fig. 1A). They belonged to four different genera, namely, norovirus, sapovirus, vesivirus, and salovirus. In addition, several contigs distantly matched unclassified members of the *Caliciviridae* family also identified in fish (Shi et al. 2016). Interestingly, several samples harbored reads belonging to different genera of the *Caliciviridae* family, suggesting co-infections. For each identified genus, we analyzed further the contigs gathering more than 100 reads, as a quality threshold to avoid contaminants or wrongly assembled contigs. For each genus, we could identify several individual contigs, sometimes in the same sample (Fig. 1B). Importantly, complete or nearly-complete genomes were identified for all genera in different samples (Fig. 1B, dotted line). We further analyzed the diversity and sub-genus classification of these long contigs, genus per genus.

### 3.2. Norovirus

Among the contigs grouping more than 100 reads, five were most closely related to a norovirus sequence using BLASTn, and also assigned to norovirus by the Norovirus typing tool 2.0 (Table 2). They were identified in three different samples with different collection dates spanning the whole study, and originating from both grey seals (FPh86) and harbor seals (FPh115, FPh127) (Tables 1 and 2). They were all longer than 7850 b, representing complete or nearly complete genomes, and were assigned to the GII or GIV genogroups by the Norovirus Typing tool 2.0, albeit with unassigned genotypes or P-types and high genetic distances. Interestingly, two samples yielded two different norovirus contigs each, indicative of co-infection (FPh86 and FPh115). To verify that these contigs were correctly assembled with adequate segregation of reads belonging to each virus, assembly was performed again with a second assembler, yielding slightly shorter but otherwise identical norovirus contigs except for a few nucleotides at the low-covered extremities (Supplementary Table S1). Variant-calling analysis on the mapping of reads to each contig showed little minority variants (Supplementary Table S1), confirming the validity of the assembly.

**Table 2:** List of NoV contigs assembled from more than 100 reads and their identification using BLASTn and the Norovirus Typing Tool 2.0, grouped by whole genomes (top half) and partial genomes (bottom half).

Sample	Contig	Size (b)	Reads	blastn hit, strain (% identity)	Norovirus typing tool (RdRp, VP1)
FPh86	m2	8020	163 178	MK067293, NoV GVI dog AN1633 (80.4)	GIV
FPh86	m3	7406	1706	MW305579, NoV GII KL137 (73.1)	GII (NA, GVIII)
FPh115	m6	7897	23 369	MW305579, NoV GII KL137 (73.1)	GII (NA, GVIII)
FPh115	m7	7813	13 957	LC509011, NoV GII swine Sw1 (73.9)	GII (NA, NA)
FPh127	m3	7735	1806	MK067293, NoV GVI dog AN1633 (80.0)	GIV

Size: size of contig after trimming extremities with coverage < 3 reads for phylogenetic analyses, in bases (b). NA, not assigned; NoV, norovirus.

**Figure 2:**
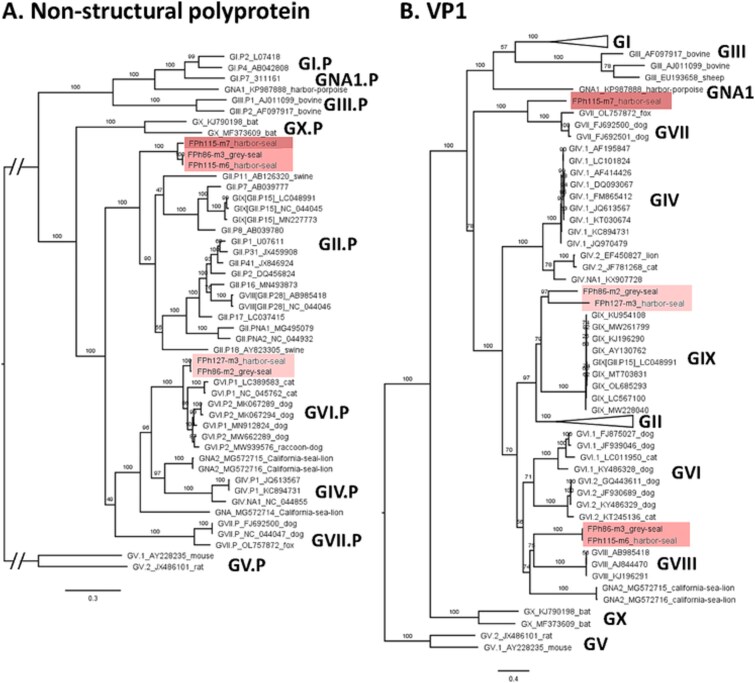
Norovirus contigs from seals form three groups related to different genogroups and P-groups. Each group is highlighted with a specific color shade in both trees. (A) Maximum-likelihood phylogenetic tree of the full non-structural polyprotein sequence translated from ORF1 of 5 contigs obtained from seals and 43 reference sequences. (B) Maximum-likelihood phylogenetic tree of the full VP1 sequence translated from ORF2 of 5 contigs obtained from seals and 184 reference sequences. Reference sequences are identified by their GenBank accession number and genogroup or P-group according to (Chhabra et al. 2019). The host from which a strain was isolated is indicated when not human. Numbers left of nodes indicate ultrafast bootstrap values. Trees were rooted using the GV murine norovirus as an outgroup.

Phylogenetic trees were constructed using sequences from the GenBank nucleotide database recommended as references (Chhabra et al. 2019), for the whole non-structural polyprotein and the whole VP1 separately (Fig. 2). Using the non-structural polyprotein sequence, the contigs segregated in two groups. One group comprised three contigs (FPh115-m6, FPh115-m7, and FPh86-m3), and was most closely related and ancestral to the GII P-group. The other group comprised two contigs (FPh86-m2, FPh127-m3) and was most closely related to the GVI P-group. Using the whole VP1 sequence, we found the contigs formed three groups, one group of two contigs (FPh115-m6 and FPh86-m3) most closely related to GVIII, one contig (FPh115-m7) most closely related to GVII, and a group of two contigs (FPh86-m2, FPh127-m3) most closely related to GIX. Interestingly, these groups gathered contigs assembled from both species of seals.

Surprisingly, the FPH115-m7 sequence was quite close to a group of two seal noroviruses (FPh115-m6, FPh86-m3) for the non-structural polyprotein, but very distant for VP1. To verify this grouping, nucleotide-based alignments and trees were also built (Supplementary Fig. S1). Identical results were obtained for the seal noroviruses with ORF1 nucleotide and amino-acid sequences. For ORF2, the overall structure of the tree is also very similar, with FPH115-m7 still closer to GVII and a group of two (FPh86-m2, FPh127-m3), closer to GIX. However, the third group (FPh115-m6, FPh86-m3) is not grouped with GVIII at the nucleotide level as it was with amino-acid sequences, but forms a group at the root of the cluster gathering GVII, GVI, GIV, GII, and GIX. Yet, the overall divergence between the different seal norovirus groups is maintained in both types of trees.

Discrepancies in the structure of the polyprotein/ORF1 versus VP1/ORF2 trees suggest an ancient recombination event between the different groups. Analysis of sequence similarity across the genome did not show clear recombination patterns for the (FPh115-m6, FPh86-m3) group and GVIII or GII, nor for the (FPh86-m2, FPh127-m3) and GIX (Supplementary Fig. S2). However, this analysis further support that FPh115-m7 could have acquired their VP1 gene from a recombination between the ancestors of GVII and that of (FPh115-m6, FPh86-m3).

### 3.3. Sapovirus

Compared to the norovirus, the sapovirus reads were less abundant (Fig. 1), but five contigs were assembled from more than 100 reads each, with a satisfactory coverage, in four samples (Table 3). Of them, three were all assigned to sapovirus GV using the Norovirus Typing tool. Two of them represented nearly whole genomes in two different samples (FPh117-m2, FPh116-m4). In a third sample, FPh114, a long contig >6000b was retrieved. A fourth sample, FPh85, yielded two long contigs, each spanning one half of the sapovirus genome. These contigs overlapped by 79 bases, albeit without paired reads, and were not joined by the assembler. They were assigned to sapovirus GVIII by the Norovirus Typing tool.

**Table 3:** List of SaV contigs assembled from more than 100 reads and their identification using BLASTn and the Norovirus Typing Tool 2.0.

Sample	Contig	Size (b)	Reads	Blast hit, strain (%id)	NoV Typing tool
FPh117	m2	7176	2058	MK291480, SaV GV 17W1094 (73.6)	SaV GV
FPh116	m4	7390	2576	MK291480, SaV GV 17W1094 (73.6)	SaV GV
FPh114	m9	6391	602	MG571784, SaV GV clone V18D (73.8)	SaV GV
FPh85	m5	2997	253	AGH15840, SaV swine WG214D^a^ (87.4)	SaV GVIII
FPh85	m3	3491	313	AB623037, SaV OH08021 (71.9)	SaV GVIII

a Hit was retrieved using DIAMOND on the GenBank protein sequence database.

Size: size of contig after trimming extremities with coverage < 3 reads for phylogenetic analyses, in bases (b). SaV, sapovirus.

**Figure 3:**
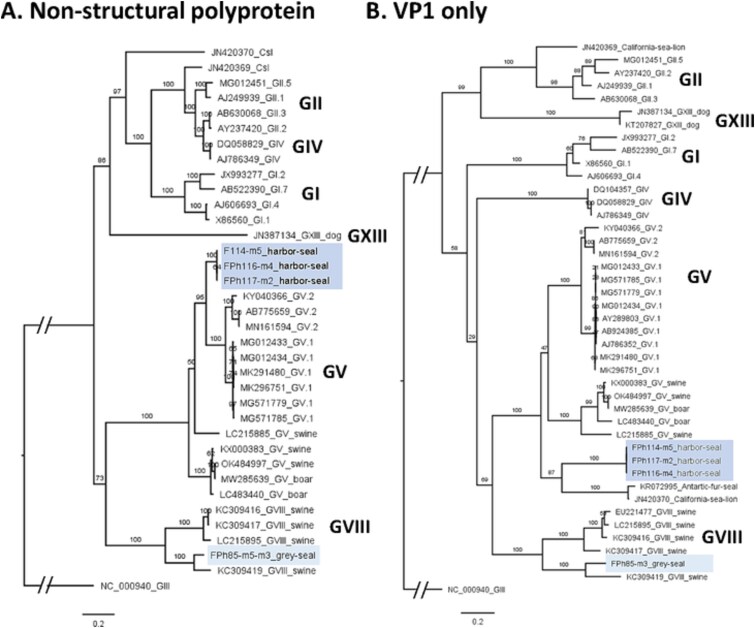
Sapovirus contigs from seals form two groups related to genogroups V and VIII. Each group is highlighted with a color shade in both trees. (A) Maximum-likelihood phylogenetic tree of the non-structural polyprotein sequences translated from ORF1 of 4 contigs obtained from seals and 31 reference sequences of known genogroups. (B) Maximum-likelihood phylogenetic tree of the full VP1 sequence translated from ORF1 of 4 contigs obtained from seals and 37 reference sequences of known genogroups. Reference sequences are identified by their GenBank accession number and genogroup according to (Oka *et al.*, Archives of Virology, 2016). The host is indicated when not human. Numbers left of nodes indicate ultrafast bootstrap values. Trees were rooted using GIII as an outgroup, according to (Oka *et al.*, Archives of Virology, 2016) and other genogroups were omitted as more distant.

Overall, the five contigs were long enough to be used for phylogenetic analyses, either on the non-structural part of ORF1, or on the VP1 sequence. In both trees, the seal sapovirus sequences formed two groups, consistent with their assignation to different genogroups by BLASTn and the Norovirus typing tool (Table 3). Using the non-structural polyprotein sequence, the first group fell within the GV, which also comprise human and swine viruses (Fig. 3A), with a high bootstrap value (95). Using the VP1 sequence, this group was most closely related to sapovirus from fur seal and sea lion, forming a “pinniped” group itself related and ancestral to the GV, supported by a high bootstrap value (Fig. 3B). Differences in tree topologies suggest an ancient genome recombination between the VP1 and the rest of ORF1 in this group. The second group, comprising the two contigs from the same sample FPh85, was most closely related to a GVIII sequence from swine using the non-structural part of ORF1. One of these two contigs did not span VP1, but the second one was also most closely related to the same GVIII using VP1 sequence only.

Genetic distances computed based on the nucleotide sequence of the whole VP1 gene, were minimal between the first group of seal sapovirus and the GV.1 references, with a mean value of 0.366 suggesting that it belongs to the GV, but not the same genotype, according to classification criteria described by Oka et al. (Oka et al. 2015). For the other sequence, FPh85-m3, closest to GVIII, the mean distance of 0.365 also points to possible new genotype within the GVIII.

### 3.4. Vesivirus

A high number of reads assigned to the vesivirus could be assembled into 16 contigs from 6 samples collected throughout the study period, from harbor seals and from 2 seals of unidentified species (Tables 1 and 4). They were assigned to the ferret badger vesivirus (VeV), the mink calicivirus or the canine calicivirus using the Norovirus typing tool. Using BLASTn, they matched vesivirus sequences from dogs, walrus, sea lion, or mink. Among them, six contigs with high coverage were near-complete genomes displaying the three expected ORF (Table 4).

**Table 4:** List of VeV contigs forming whole genomes and their identification using BLASTn and the Norovirus Typing Tool 2.0.

Sample	Contig	Size (b)	Reads	Blast hit, strain (% identity)	NoV typing tool
FPh117	m1	8664	196 296	GQ475301, calicivirus Allston 2009/US (74.7)	VeV ferret badger vesivirus
FPh115	m5	8655	35 653	GQ475303, calicivirus Geel 2008/Belgium (74.8)	VeV mink calicivirus
FPh116	m2	8480	26 620	MF327135, canine calicivirus A128T (73.8)	VeV ferret badger vesivirus
FPh114	m3	8633	14 377	MF327135, canine calicivirus A128T (74.1)	VeV ferret badger vesivirus
FPh112	m0^a^	8340	40 614	MF327135, canine calicivirus A128T (73.4)	VeV mink calicivirus
FPh113	m0^a^	8284	13 665	JN204722, canine vesivirus Bari/212/07/ITA (75.3)	VeV mink calicivirus

a Sequence assembled from six overlapping contigs and by mapping.

Size: size of contig after trimming extremities with coverage < 3 reads for phylogenetic analyses, in bases (b). VeV, vesivirus.

**Figure 4:**
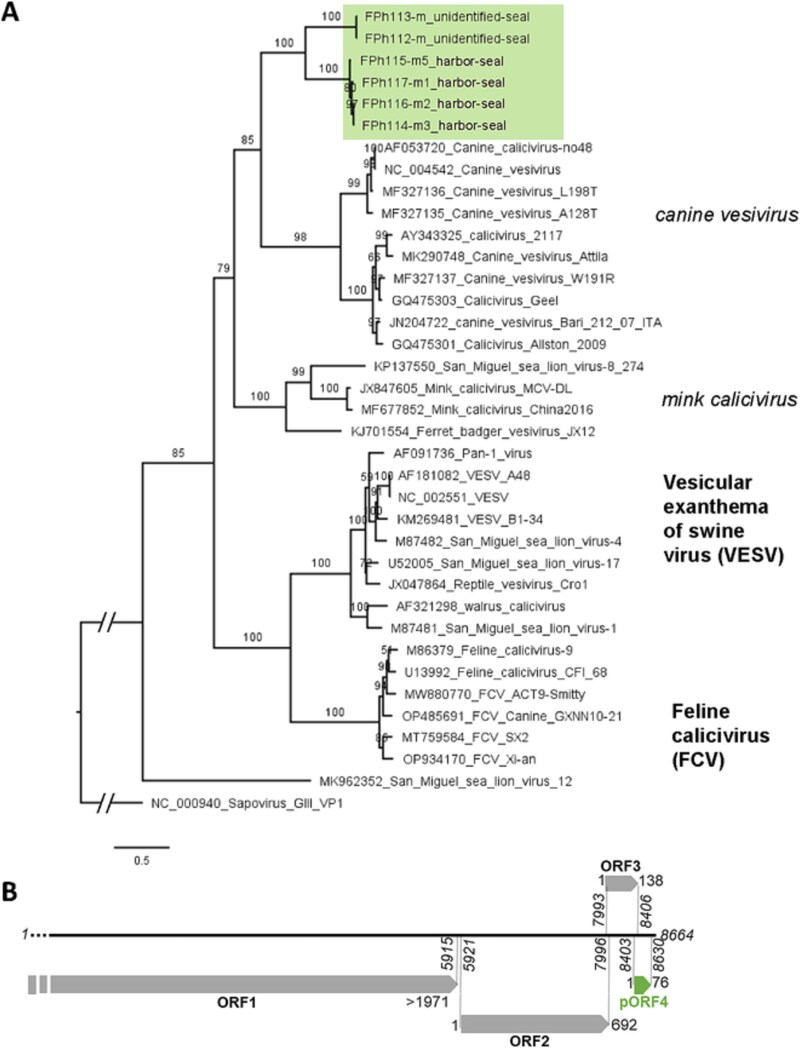
Vesivirus contigs from seals form a monophyletic group related to the canine vesivirus. (A) Maximum-likelihood phylogenetic tree of the whole VP1 sequences translated from ORF2 of 6 genome sequences obtained from seals (color shade) and 31 reference sequences identified by GenBank accession number, virus name and strain when relevant. Numbers left of nodes indicate ultrafast bootstrap values. One Sapovirus GIII VP1 sequence was used as an outgroup to root the tree. Vesivirus regroup two recognized species, vesicular exanthema of swine virus (VESV) and FCV (bold upper case), and additional unclassified groups (italic lower case) or strains. (B) Genomic organization of the Vesivirus from seals, based on sequence FPh117-m1, with the putative ORF4 observed in 4 contigs.

Near-complete genomes were used for phylogenetic analyses together with sequences retrieved from the Genbank database and belonging to different vesivirus species and clusters (Supplementary Fig. S3). More VP1 sequences being available, especially for San Miguel sea lion viruses, we also constructed a phylogenetic tree on the full VP1 protein sequence (Fig. 4A). In both trees, the seal vesivirus formed a monophyletic group, most closely related to the canine calicivirus and canine vesivirus, but with a high genetic distance. They were not grouped with other sequences from pinnipeds such as the San Miguel sea lion virus 1, 4, 8, or 12 or the walrus calicivirus which were spread throughout the tree.

Interestingly, for the four sequences with a complete ORF3, including two genomes sequenced up to the polyA tail, we observed an unusually long 3′ UTR after ORF3 (up to 240 b). It comprised a conserved, putative fourth ORF of 228 b (76 amino-acids) between an ATG start codon and a TAG stop, in −1 frameshift and four bases overlap with the ORF3 (Fig. 4B), similar to the structure of the junction between ORF2 and 3. Searching for homologs of this ORF and putative protein sequence using BLASTn and BLASTp did not yield any hit, and no conserved domain was detected using the CD-search tool.

### 3.5. Salovirus and unassigned fish viruses

Beside the three genera of mammal-infecting caliciviruses, many reads were assigned to the salovirus which are known to infect Atlantic salmon. We retrieved 19 contigs assembled from more than 100 of these reads, in 11 samples collected across the study period and from the 2 species of seals. All matched the Atlantic salmon calicivirus (ASCV), the unique species of salovirus, using both BLASTn and the Norovirus typing tool. Eleven contigs longer than 6000 b are listed in Table 5. The four longest were complete genomes, with a typical salovirus organization comprising a 20b 5′ untranslated region, a long ORF1 encoding the putative non-structural polyprotein and VP1, and a short ORF2 overlapping with ORF1 3′ end. One contig (FPh125-m2) missed the beginning of ORF1 but comprised the complete ORF2. The six last missed both the beginning and the end of ORF1, and ORF2. In addition, eight shorter salovirus contigs were assembled from more than 100 reads but not used for phylogenetics (Table 5).

**Table 5:** List of salovirus contigs assembled from more than 100 reads and their identification using BLASTn.

Sample	Contig	Size (b)	Reads	Blast hit, strain, (%identity)
FPh85	m1	7415	30 401	KJ577140, Atlantic salmon calicivirus AL V901 (74.2)
FPh128	m8	7394	35 744	KJ577140, Atlantic salmon calicivirus AL V901 (86.3)
FPh126	m2	7379	11 250	KJ577140, Atlantic salmon calicivirus AL V901 (86.3)
FPh127	m4	7386	4576	KJ577140, Atlantic salmon calicivirus AL V901 (86.3)
FPh125	m2	6735	2921	KJ577140, Atlantic salmon calicivirus AL V901 (85.4)
FPh86	m5	6614	17 140	KJ577140, Atlantic salmon calicivirus AL V901 (74.4)
FPh86	m6	6614	16 665	KJ577140, Atlantic salmon calicivirus AL V901 (74.0)
FPh116	m6	6482	49 402	KJ577140, Atlantic salmon calicivirus AL V901 (73.6)
FPh116	m7	6476	10 792	KJ577140, Atlantic salmon calicivirus AL V901 (74.0)
FPh114	m6	6472	16 006	KJ577140, Atlantic salmon calicivirus AL V901 (73.6)
FPh114	m7	6478	3626	KJ577140, Atlantic salmon calicivirus AL V901 (74.0)
FPh115	m25	5362^a^	139 703	KJ577140, Atlantic salmon calicivirus AL V901 (73.7)
FPh115	m26	5362^a^	21 004	KJ577140, Atlantic salmon calicivirus AL V901 (74.2)
FPh115	m174	1638^a^	50 040	KJ577140, Atlantic salmon calicivirus AL V901 (77.3)
FPh120	m217	1127^a^	141	OP933700, MAG: Fish-associated calicivirus ft105cal (74.1)
FPh120	m279	1027^a^	156	KJ577140, Atlantic salmon calicivirus AL V901 (82.3)
FPh114	m556	695^a^	1356	KJ577140, Atlantic salmon calicivirus AL V901 (84.0)
FPh116	m625	694^a^	4423	KJ577140, Atlantic salmon calicivirus AL V901 (84.0)
FPh115	m1866	621^a^	6309	KJ577140, Atlantic salmon calicivirus AL V901 (82.1)

a Size of raw contigs as yielded by the assembler.

Size: size of contig in bases (b), after trimming extremities with coverage < 3 reads for phylogenetic analyses except when^a^.

**Figure 5:**
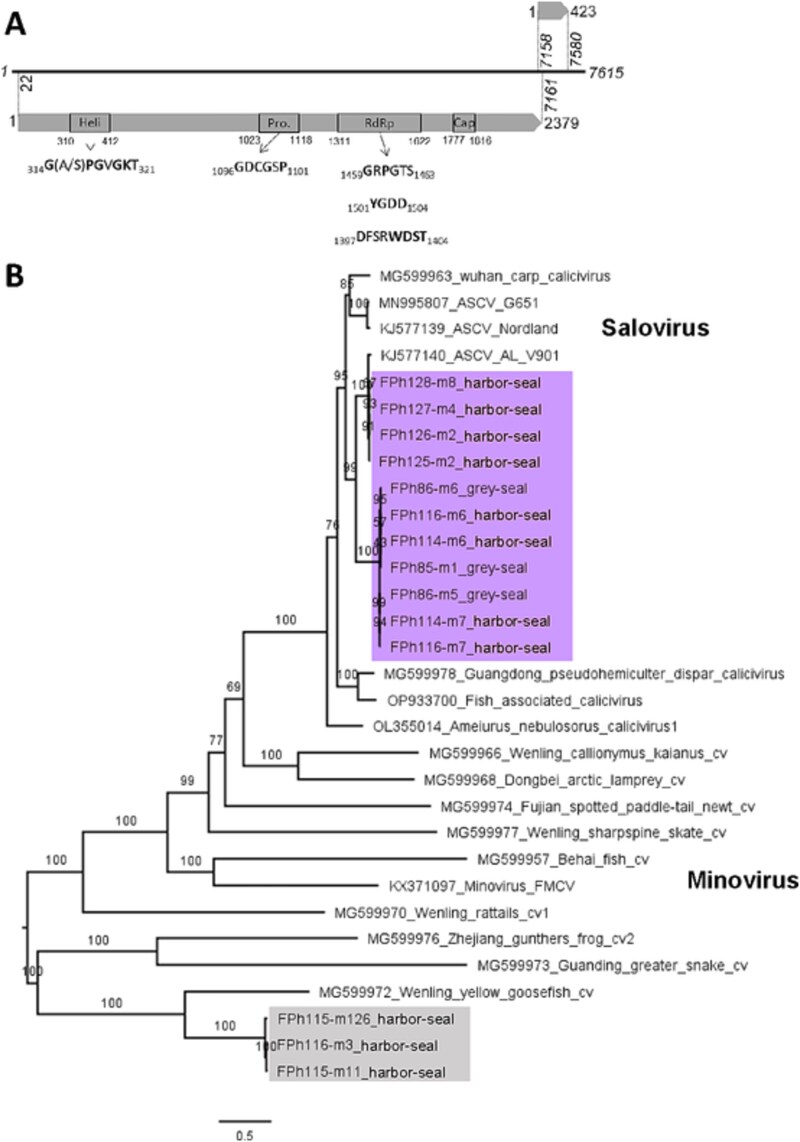
Viral contigs related to fish calicivirus. (A) Genomic organization of the unclassified *Caliciviridae* from seal feces based on FPh116-m3 contig. The nucleic acid sequence (black line, positions in italic) encodes two open reading frames (grey arrows). The first ORF comprise conserved domains identified as helicase (Heli.), 3C-like viral protease (Pro.), RNA-dependent RdRp and Calicivirus major capsid protein (Cap.) and corresponding motifs observed in all *Caliciviridae* listed below the arrows. (B) Maximum-likelihood phylogenetic tree of the sequence translated from ORF1 of 14 contigs obtained from seals and 17 sequences of calicivirus (cv) from fish and reptiles, identified by their GenBank accession number and name. The host is indicated when not stated in the virus name. Numbers left of nodes indicate ultrafast bootstrap values. The tree is rooted to its midpoint. Fish calicivirus regroup two recognized genera, salovirus and minovirus (bold case) and additional unclassified sequences. Eleven sequences from seal feces (purple) are grouped within the Salovirus, close to the unique viral species of this genus, the ASCV. Three other sequences form a monophyletic group (grey) close to a calicivirus identified in yellow goosefish.

In addition, we found 9 contigs assembled from more than 100 reads, distantly matching calicivirus sequences previously identified in fish, in 6 samples also from the 2 species of seals (Table 6). The longest (FPh116-m3) corresponded to a whole genome and displayed a long ORF1 and a short ORF2 with four nucleotides overlap and a +2 frameshift (Fig. 5A). The polyprotein sequence translated from the ORF1 displayed conserved domains and motifs characteristic of the *Caliciviridae* (Fig. 5A) (Mikalsen et al. 2014). Two other contigs corresponded to nearly complete genomes of calicivirus, with a long but incomplete ORF1 and missing ORF2. The six other contigs were shorter partial genomes, covered by less reads (Table 6).

**Table 6:** List of unassigned calicivirus contigs assembled from more than 100 reads and their identification.

Sample	Contig	Size (b)	Reads	Best hit	% id.	e-value	Match length
FPh116	m3	7615	7886	Wenling yellow goosefish calicivirus^a^	43.4	0.0e+00	2297
FPh115	m11	7132	328 429	Wenling yellow goosefish calicivirus^a^	43.3	0.0e+00	2294
FPh115	m12	7175	10 880	Wenling yellow goosefish calicivirus^a^	43.2	0.0e+00	2295
FPh120	m18	3223^b^	681	Wenling yellow goosefish calicivirus^a^	41.7	1.0e-227	988
FPh120	m24	2866^b^	785	Wenling yellow goosefish calicivirus^a^	45.6	3.5e-250	935
FPh114	m44	2491^b^	216	Calicivirus Mystacina/New Zealand/2013/3H^a^	38.8	2.6e-21	139
FPh121	m22	2364^b^	123	Wenling yellow goosefish calicivirus^a^	46	1.0e-222	761
FPh120	m78	1678^b^	374	Wenling yellow goosefish calicivirus^a^	44	3.3e-95	407
FPh115	m1227	770^b^	639	Wenling yellow goosefish calicivirus^a^	59.7	7.9e-92	308

a Hit was retrieved using DIAMOND on the GenBank protein sequence database.

b Size of raw contigs as yielded by the assembler.

Size: size of contig after trimming extremities with coverage < 3 reads for phylogenetic analyses, except when^b^. % id., identity percentage to the blast hit.

To better identify these potential fish-infecting caliciviruses, we constructed a phylogenetic tree on the ORF1 protein sequence using fish, reptile or amphibian calicivirus from the Genbank database, including reference sequences from the salovirus and minovirus genera (Fig. 5B). The 11 long contigs identified as salovirus formed two groups, indeed falling within the salovirus. The three longest contigs corresponding to unclassified fish calicivirus formed a monophyletic group, most closely related to the Wenling yellow goosefish calicivirus as observed using DIAMOND, among the other caliciviruses from fish or other unclassified sequences.

## 4. Discussion

### 4.1. Diversity of caliciviruses from seal feces and their hosts

Using samples of seal feces collected in SPM within a sampling period of 1 year, we identified complete or nearly-complete genomes belonging to the *Caliciviridae* family, classified within four known genera (norovirus, sapovirus, vesivirus, and salovirus) or belonging to an unclassified, distantly related group of fish viruses (Fig. 1, Fig. 5). The norovirus, sapovirus, and vesivirus sequences were interpreted as resulting from seal infection by the respective viruses, which are known to infect mammals. The seal sapovirus were indeed more closely related to strains identified in other Pinnipeds, the California sea lion and Antartic fur seal (Martínez-Puchol et al. 2022). Similarly, one group of seal norovirus and the seal vesivirus were more closely related to viruses from dogs, which belong to the same order, the Carnivora. More surprisingly, the other groups of seal norovirus were more closely related to human viruses (GVIII, GIX). Proximity between norovirus from humans and marine mammals was already observed with the harbor porpoise norovirus (GNA1) that is related to GI (Graaf et al. 2017). This suggest that further sampling of marine mammal species could allow to better understand the origin and evolution of human norovirus.

Conversely, the origin of calicivirus sequences previously identified in fish is less straightforward. Salovirus sequences were retrieved from 8 samples out of 22, showed a high similarity one with another and fell within the group of ASCV sequences with high bootstrap values (Fig. 5). ASCV itself was isolated from infected Atlantic salmon and could be cultured *in vitro* in salmon cells (Mikalsen et al. 2014), which proves that this is indeed a salmonid virus. ASCV was identified recently in Atlantic salmons farmed in both Atlantic and Pacific Canada (Mordecai et al. 2021), which confirms it presence in the Atlantic area of Saint-Pierre-et-Miquelon. Moreover, seals from the North Atlantic are known to prey on salmon (Butler et al. 2011, Lenky and Sjare 2011). This strongly suggest that the salovirus sequences obtained here were acquired by seals from their feeding of salmonids, and likely do not represent a true infection, despite the high number of reads. The fifth group of *Caliciviridae* identified in our study were also closely related one to each other, their closest relative in databases being a sequence obtained by metagenomic analysis of yellow goosefish tissues, without isolation of the corresponding virus. Given their higher proximity to *Caliciviridae* sequences from other fish, amphibian, and reptiles, these sequences are nonetheless more likely diet-derived, and we considered them as fish viruses based on the phylogenetic analysis (Fig. 5). Diet-derived viruses were previously observed in feces samples of Antartic fur seals using metagenomics (Martínez-Puchol et al. 2022). This highlight that applying viromics on predator species allows to unravel a wider diversity of viruses, encompassing also those of prey. However, linking virus and host is rendered more difficult.

### 4.2. Circulation of caliciviruses in the seal populations

Mammalian genera of *Caliciviridae* family (norovirus, sapovirus, vesivirus) were detected in the two studied species of seals, and at all study periods. Interestingly, for norovirus, very similar sequences were obtained from grey seals and harbor seals (for instance, FPh86-m3 and FPh115-m6) sampled a few months apart, but also after 1 year (FPh86-m2, sampled in May 2019 and FPh127-m3, in June 2020). For sapovirus, we observed a group of sequences sampled from harbor seals within 20 days, and a distant sequence from a grey seal, sampled a few months before. Finally, for vesivirus, all long sequences were obtained from harbor seals or unidentified seals, sampled in the summer and fall of 2019. This suggests that while norovirus circulate in both seal species and can persist in their populations for over 1 year, the sapovirus and vesivirus strains appear more specific and less persistent. Of note, cross-species transmission of vesivirus were reported previously from marine mammals (Smith et al. 1998), and our study, limited to 22 samples, cannot rule out that these strains are actually able to infect other sea mammal species. Since feces were collected from unidentified individual seals, we also cannot rule out that some samples taken at different days originate from the same animal.

Finally, aside from the likely diet-acquired fish viruses, several samples showed a co-infection of seals with mammalian viruses from different genera, or from different groups within the same genus. Sample FPh115 for instance yielded two different whole genomes of norovirus and one of vesivirus. Together, our results show that the SPM seals are a reservoir of diverse *Caliciviridae*, that can circulate for more than a year and across two seal species. Whether this is also true for other seal populations, from other species or other areas, remains to be assessed. The pathogenic potential of these viruses for seals also remains unknown. No obvious signs of disease were observed in the sampled population. Other *Caliciviridae* were identified in apparently healthy animals, such as hare infected with newly identified lagoviruses (Mahar et al. 2019).

### 4.3. Phylogenetics and classification of caliciviruses from seal feces

Phylogenetic studies conducted on the putative protein sequences translated from (nearly-)whole viral genomes showed that the seal viruses formed distinct groups, often distantly related to known genogroups or species. The diversity was highest for the norovirus, with three groups of sequences related to different genogroups. Given the large genetic distance (40%–65% identity) between these groups and the known norovirus genogroups, these may represent new genogroups, or at least new genotypes or P-types. Furthermore, there was a marked difference in tree topologies using the polyprotein/ORF1 or the VP1/ORF2 sequences of seal norovirus, suggesting ancient recombination events between the ancestors of the different groups. Analysis of genetic similarity across the genome did not show clear recombination patterns for the (FPh115-m6/FPh86-m2) and (FPh86-m3/FPh127-m2) groups, possibly because the parental strain for the ORF2 remains unknown. Conversely, the FPh115-m7 sequence could result from an ancient recombination with GVII. GIV.2, GVIII, and GIX viruses are known to segregate independently of their respective polymerase sequences (GVI.P, GII.P28 and GII.P15, respectively) (Chhabra et al. 2019). Interestingly, the seal noroviruses are related to these P.groups and/or genogroups, and could help understand the evolutionary history of noroviruses.

For sapovirus, a group of three seal sequences forms a possible new genotype within the GV, and another sequence could belong to the GVIII, based on VP1 nucleotide sequence alignments and the distance criteria described in (Oka et al. 2015). In a recent study, a new classification scheme was proposed for sapovirus, with a dual typing similar to the norovirus, based on amino acid sequence alignments of RdRp and VP1 (Zhao et al. 2024). Here, applying their criteria, the first group of seal sapovirus also belong to GV for VP1 with mean distances of 0.389, and the FPh85-m3, to GVIII, with a mean distance of 0.323. However, given the small reference dataset used in our study, these genetic distances are an estimate. Future classification work will be needed to verify the assignation of the seal sapovirus sequences to putative new genotypes.

High genetic distances were also observed between the seal vesivirus sequences and their closest relative, canine vesivirus (62%–64% identity on VP1), which could make them a separate species. Surprisingly, we detected a conserved open reading frame at the 3′ of the complete Seal vesivirus genomes. It displays the same −1 frameshift and 4 nt overlap with ORF3 as the junction between ORF2 and ORF3, and possibly codes for a 76 amino-acids protein. However, the absence of conserved domains or relatives in the current databases precludes any hypotheses about its potential function. Several vesivirus reference genomes, such as VESV, feline calicivirus, canine calicivirus, or walrus calicivirus, also show a long 3′UTR of about 200 bp, but devoid of any ORF of this size. Anyways, more sequences, and sampled in other locations, are needed to confirm these putative new phyla and their characteristics.

Conversely, salovirus sequences from seal feces clearly fell within the ASCV species, as outlined earlier, and expand the known diversity of this poorly studied genus. Finally, the unclassified and divergent fish Calicivirus we describe here present all the genomic characteristic of *Caliciviridae*, including conserved protein domains. This finding suggests that a high diversity of *Caliciviridae* remains to be uncovered in fish.

### 4.4. Zoonotic potential of seal viruses

Seal haul-out sites can result in large amounts of feces deposited in coastal waters, such as recreational areas, which can lead to poor water quality and beach closures (Paar et al. 2024). This study was primarily undertaken to assess the possible impact of growing seal populations on the microbiological quality of the coastal area in SPM, their possible negative impact on bathing and shellfish-harvesting activities locally and the risks they may pose to human populations (Godino Sanchez et al. 2024). Norovirus and sapovirus in particular are known human pathogens. Here, some of the norovirus and sapovirus sequences from seals were more closely related to human genogroups (norovirus GIX, sapovirus GV), but with high distances, suggesting that cross-species transmission is unlikely. However, further analyses are necessary to completely rule out the risk for human health. The binding patterns of these seal viruses on human cells or tissues may help to assess the possibility of a host jump (Villabruna et al. 2020). Assessing possible exposure scenarios, such as shellfish contamination through filter-feeding in seawater contaminated with seal feces, followed by their consumption by humans, is also warranted.

## 5. Conclusion

In conclusion, beyond the detection of high *Escherichia coli* concentrations and bacterial genera including human pathogens (Godino Sanchez et al. 2024), this study reveals the presence of new viral sequences in seal feces from SPM, some related to human viruses. These new members of the *Caliciviridae* family, especially from the norovirus, sapovirus, vesivirus, and salovirus genera were observed in two major species of seals from the North-Western Atlantic. These results expand the knowledge on the diversity and evolution of this clinically relevant viral family, and will allow the design of molecular tools to further assess their geographical repartition, zoonotic potential, and the exposure of the human population.

## Supplementary Material

Supplementary_materials_veag029

## Data Availability

Raw sequencing data and genomes are available in the European Nucleotide Archive (ENA) database (Project: PRJEB64019; Study: ERP149173; Samples from ERR11734880 to ERR11734912 and ERR13601248 to ERR13601276) and at https://sextant.ifremer.fr/eng/Data/Catalogue#/metadata/76beca06-ac02-49ce-810e-94f9c121e868
