# Supplementary material for: Metaviromics reveals a high diversity of viruses belonging to the Caliciviridae family in seal feces
Source: Virus Evol. 2026 May 7;12(1):veag029. doi: 10.1093/ve/veag029 (PMC13200540; doi:10.1093/ve/veag029)
Supplement: Supplementary_materials_veag029 [file supplementary_materials_veag029.zip › Supplementary_materials_veag029_Table 1.docx]

**Supplementary Table 1. Verification of the assembly of norovirus contigs in co-infected samples.**

| Contig | Length with metaSpades* | Length with Megahit* | Identity (%) | Variants¤ |
| --- | --- | --- | --- | --- |
| FPh115-m6 | 7929 | 7925 | 100 | 34 |
| FPh115-m7 | 7854 | 7834 | 100 | 31 |
| FPh86-m2 | 8069 | 7993 | 100 | 7 |
| FPh86-m3 | 7854 | 7920 | 99.14 | 7 |

* length of raw contig obtained by each assembler ; ¤ number of minority variants with frequency above 3% detected on the metaSpades contigs with mapped reads.
