# Supplementary material for: Metaviromics reveals a high diversity of viruses belonging to the Caliciviridae family in seal feces
Source: Virus Evol. 2026 May 7;12(1):veag029. doi: 10.1093/ve/veag029 (PMC13200540; doi:10.1093/ve/veag029)
Supplement: Supplementary_materials_veag029 [file supplementary_materials_veag029.zip › Supplementary_materials_veag029_figures.pdf]

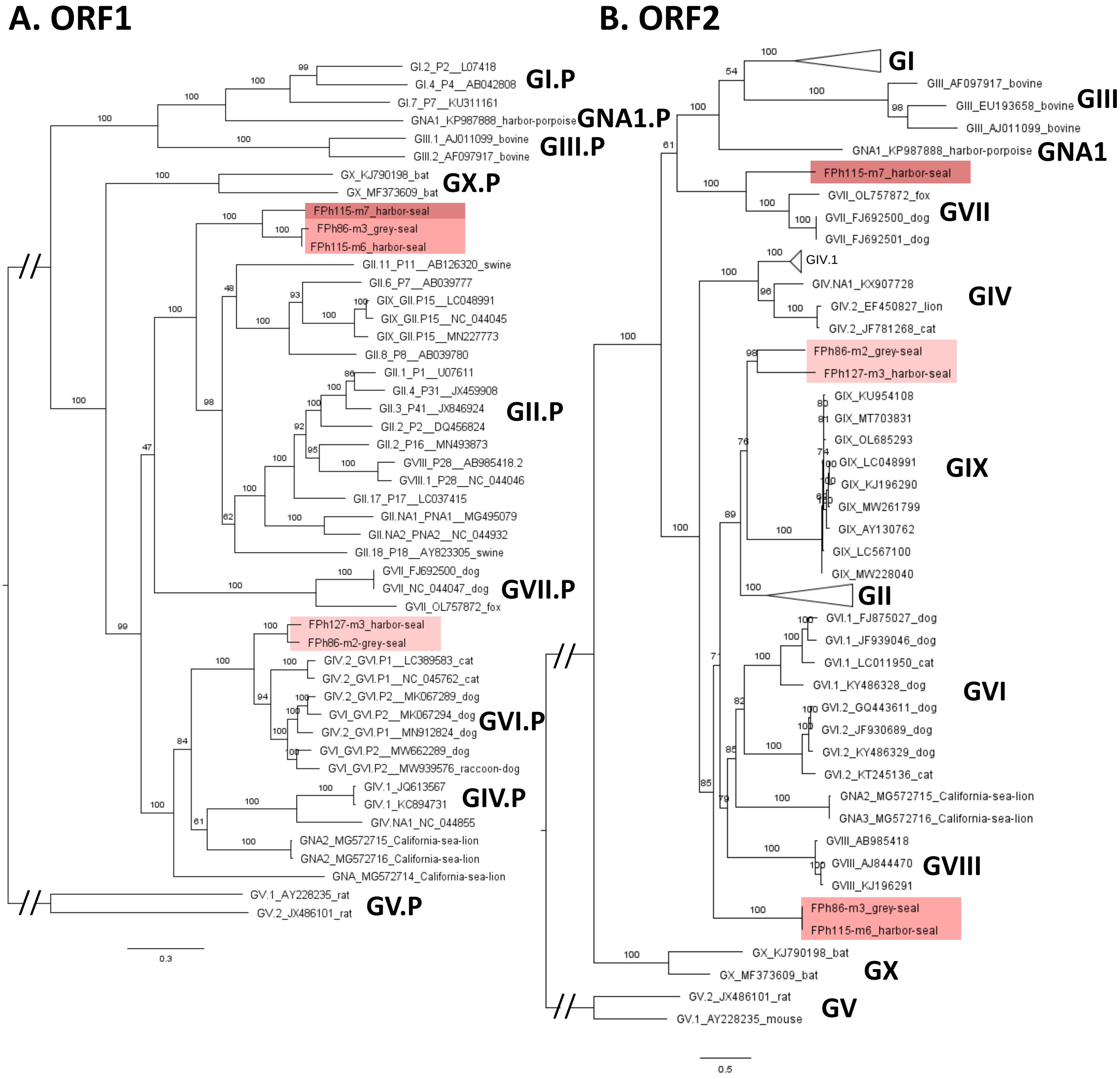

Supplementary Figure 1. Phylogenetic analysis of seal norovirus contigs based on nucleotide sequences.

A. Maximum-likelihood phylogenetic tree of the full ORF1 sequence of 5 contigs obtained from seals and 43 reference sequences.

B. Maximum-likelihood phylogenetic tree of the full ORF2 sequence of 5 contigs obtained from seals and 175 reference sequences.

Reference sequences are identifed by their GenBank accession number and genogroup or P-group according to (Chhabra et al, Journal of General Virology, 2019). The host from which a strain was isolated is indicated when not human. Numbers left of nodes indicate ultrafast bootstrap values. Trees were rooted using the GV murine norovirus as an outgroup. For both trees, the three groups of seal norovirus are highlighted with a specific shade of red.

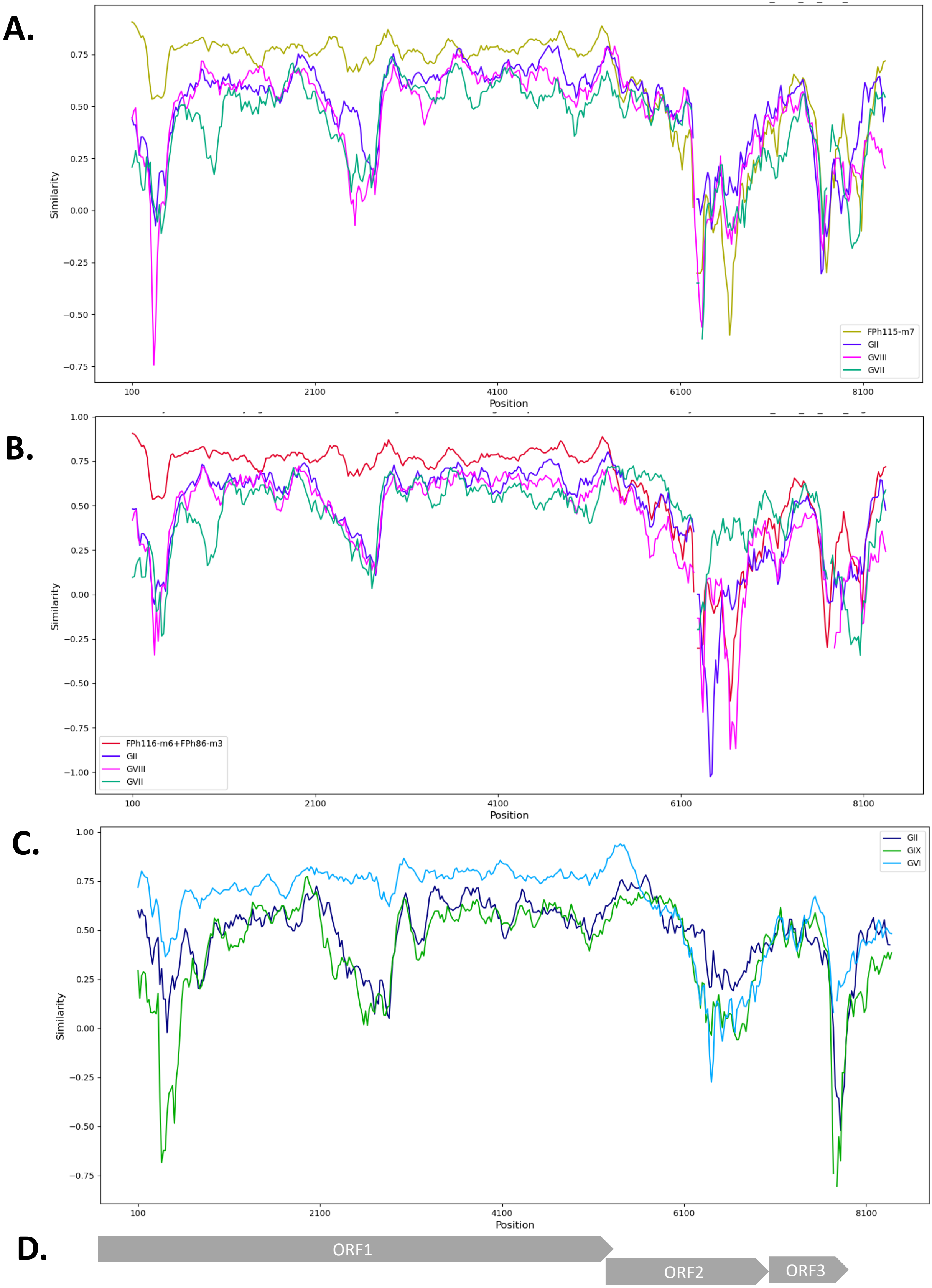

Supplementary Figure 2. Analysis of possible recombination patterns in seal norovirus genomes.

A. B. SimPlot results of complete genome alignments of FPh115-m6 + FPh86-m3 (red line), FPh115-m7 (yellow line), norovirus GII[GII.P] (AY772730, JX459908, AY823305, AB126320 – blue line), GVIII[GII.P28] (NC_044046, AB985418 - pink line) and GVII (FJ692500, FJ692501, OL757862 – green line) with respect to **A.** (FPh115-m6 + FPh86-m3), **B.** FPh115-m7. **C.** SimPlot results of complete genome alignments of FPh86-m2 + FPh127-m3, norovirus GVI[GVI.P] (MW939576, MW662289 – light blue line), GII[GII.P] (AY772730, JX459908, AY823305, AB126320 – dark blue line) and GIX[GII.P15] (MW261799, LC048991, OL685293, MT703831 – green line) with respect to (FPh86-m2 + FPh127-m3). Parameters of Simplot++ were window : 200bp, step : 20bp, model : Tamura.

D. Schematic diagram of ORF positions in the seal norovirus sequences

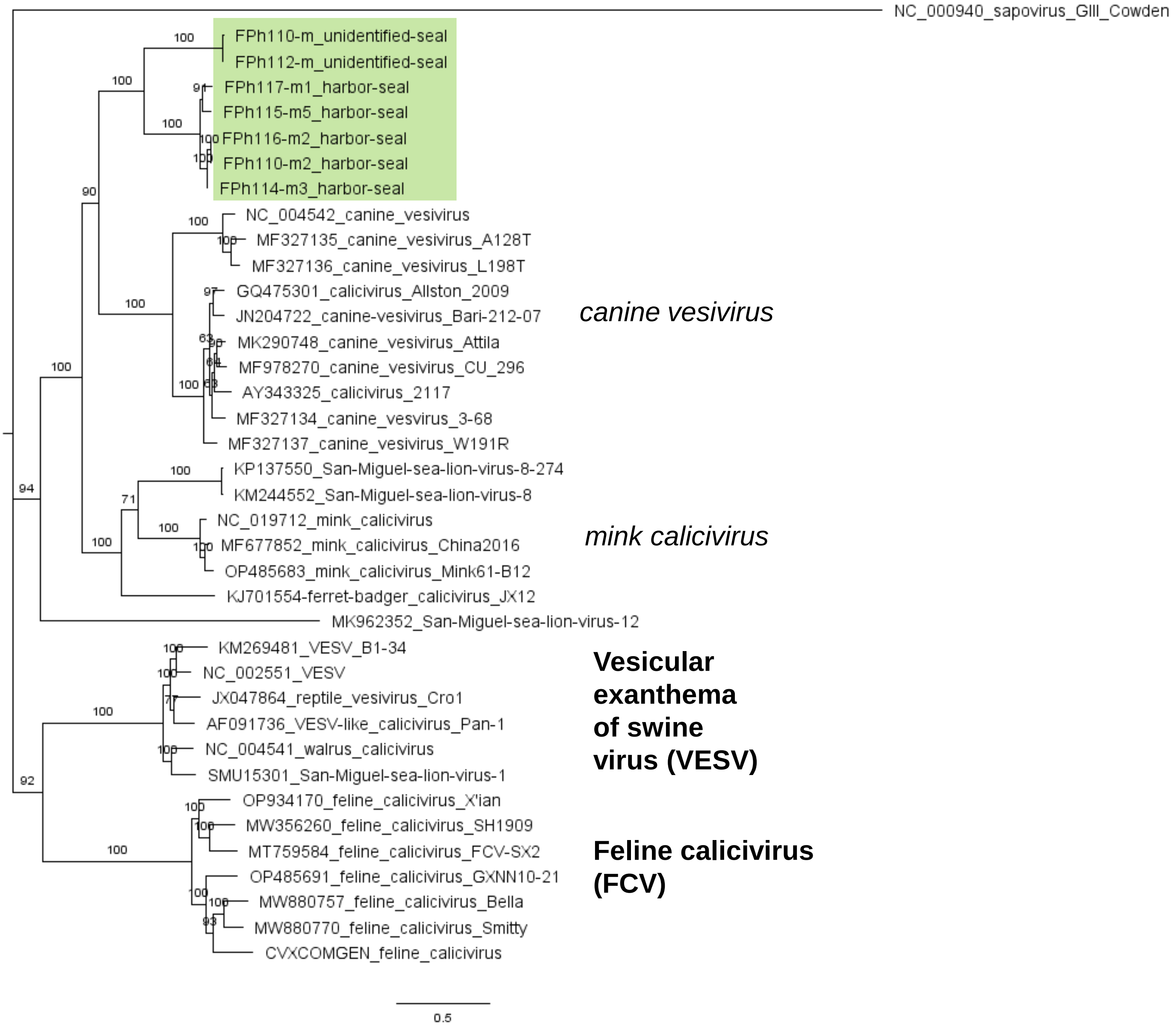

Supplementary Figure 3. Phylogenetic analysis of seal vesivirus contigs based on nucleotide sequences. Maximum-likelihood phylogenetic tree of the full genomic sequence of 7 contigs obtained from seals (green shade) and 31 reference sequences.

Reference sequences are identifed by their GenBank accession number and name. Numbers left of nodes indicate ultrafast bootstrap values. A Sapovirus GIII VP1 sequence was used as an outgroup to root the tree. Vesivirus regroup two recognized species, vesicular exanthema of swine virus (VESV) and feline calicivirus (FCV) (bold upper case), and additionnal unclassified groups (italic lower case) or strains.
